# The impact of physical activity on social anxiety among college students: the chain mediating effect of social support and psychological capital

**DOI:** 10.3389/fpsyg.2024.1406452

**Published:** 2024-06-18

**Authors:** Yiran Deng, Xianliang Wang

**Affiliations:** School of Physical Education, Shandong University, Jinan, China

**Keywords:** physical activity, college students, social support, psychological capital, social anxiety, chain mediation

## Abstract

**Objective:**

As a topic of widespread concern in the fields of mental health and public health, social anxiety has many negative impacts on the physical and mental health of contemporary college students. Therefore, this study aims to provide new ideas for solving the problem of social anxiety among college students by exploring the potential mediating role of social support and psychological capital in the relationship between physical activity and social anxiety.

**Methods:**

A cross-sectional survey was conducted on 874 college students from five universities in Shandong Province. Various self-report tools such as physical activity rating scale, social support scale, positive psychological capital scale, and social anxiety scale were used to collect information needed for this study. Related data. Use this to conduct descriptive statistical analysis, correlation analysis, and mediation effect analysis.

**Results:**

The results showed that physical activity was significantly positively correlated with college students’ social support (*r* = 0.354, *p* < 0.01) and psychological capital (*r* = 0.448, *p* < 0.01), and social support was significantly positively correlated with psychological capital (*r* = 0.556, *p* < 0.01), the above three are significantly negatively correlated with social anxiety (*r* = −0.326, −0.381, −0.432, *p* < 0.01); the mediation effect analysis shows that physical activity has a significant direct effect on college students’ social anxiety The effect value is −0.136, accounting for 43.31% of the total effect; social support and psychological capital both play a separate mediating role between physical activity and social anxiety among college students, with effect values of −0.064 and −0.073 respectively, accounting for the total effect. 20.38, 23.25%, and also played a chain intermediary role, with an effect value of −0.041, accounting for 13.05% of the total effect.

**Conclusion:**

Current research shows that physical activity can not only directly reduce social anxiety among college students, but also indirectly alleviate social anxiety among college students by increasing the level of social support and psychological capital. This has important reference significance for helping college students in China and even around the world overcome social anxiety.

## 1 Introduction

Social anxiety refers to an overt and extreme fear of embarrassment or negative evaluation during social interactions (Watson and Friend, 1969; Heimberg et al., 1999). Social anxiety will not only have a negative impact on an individual’s study, life and normal social interactions, but also lead to the complication of other mental disorders, seriously damaging an individual’s quality of life and life and health (Li et al., 2020a). It is the third most common cause of illness after depression and alcohol dependence. Mental disorders are common, with lifetime prevalence rates as high as 12% (Stein et al., 2017). For college students who are in the adaptive transition stage from teenagers to adults, they not only need to face the psychological impact of identity change, but also need to properly coordinate the cross-balance between academics, life and interpersonal relationships, and pressure from many aspects And the numerous social evaluation issues in college life may make college students more likely to have real or imagined fears of embarrassment, leading to frequent social anxiety symptoms (Russell and Topham, 2012). According to relevant surveys, about 10–33% of college students have symptoms of social anxiety, and this rate is increasing year by year (Russell and Shaw, 2009; Russell and Topham, 2012; Zhang et al., 2020). Previous studies have begun to focus on overall psychological health issues among college students, including social anxiety, but only a few have conducted in-depth investigations into the developmental trajectories of anxiety, stress, and related issues among college students (Liu et al., 2023). Currently, research on the psychological health of Chinese college students has started to use longitudinal data to elucidate causal relationships and trends. This not only clarifies that psychological issues such as anxiety, stress, and depression among college students are dynamic processes with heterogeneity among different groups but also reveals the significant role of factors such as social support and self-esteem in addressing these psychological issues among college students (Liu et al., 2023; Cao and Liu, 2024). Although the aforementioned studies have made some contributions to preventing and overcoming psychological health issues among college students, their focus has been primarily on internal individual factors. Therefore, continuing to explore the psychological factors influencing college students’ social anxiety and finding effective external means to reduce their social anxiety remains a pressing and focal issue that needs attention and resolution. Therefore, continue to explore the psychological factors affecting college students’ social anxiety and to find effective external means to effectively reduce their social anxiety are still the focus of urgent attention and solution at present.

### 1.1 Physical activity and social anxiety

As an effective means to promote the mental health development of college students, physical activity has a certain effect on alleviating social anxiety (Schumacher Dimech and Seiler, 2010). The factors that affect college students’ social anxiety are relatively complex. Previous studies have mainly explored and analyzed the role of individual cognitive factors such as peer relationships and subjective well-being on social anxiety (Holt and Espelage, 2005; Shang et al., 2023) Although these factors are important, practical and effective flexible intervention cannot be carried out. This is This limits the generalizability of the research results to some extent. However, more and more scholars have noticed that it is not enough to focus only on the impact of individual cognitive factors on social anxiety. According to the social cognitive behavioral model and self-decision-making theory, physical activity can meet the basic psychological needs of individuals and improve individual cognitive level to ensure their mental health development, which provides effective evidence for physical activity as an external factor to alleviate social anxiety (Crick and Dodge, 1994; Brunet and Sabiston, 2009). A study shows that the amount of exercise can significantly affect students’ social anxiety levels, confirming that physical activity can be an effective means to prevent social anxiety (Brunet and Sabiston, 2009). A recent study also showed that using physical activity as the main intervention method will significantly reduce the level of social anxiety in college students (Zhang and Liu, 2017). In view of this, the above studies provide reasonable evidence for physical activity to prevent and alleviate social anxiety, but the underlying mechanism of how physical activity affects social anxiety remains to be explored. Therefore, this study proposes the first hypothesis:

*H1*: Physical activity can significantly and negatively predict social anxiety among college students.

### 1.2 The mediating role of social support

Social support refers to the support provided by the outside world to help individuals overcome the stress they face (Lubis et al., 2022). Social support comes from many aspects, including family support, material support, spiritual support, peer support, etc. A good level of social support will help individuals build strong self-confidence and encourage individuals to reasonably handle various events in daily life and carry out effective activities. Social interactions. Relevant research shows that social support is an important factor in improving an individual’s perception of the controllability of events. The definition of social anxiety emphasizes that uncontrollability and negative evaluation of environmental changes are the main causes of individual anxiety, and perceived social support provides important help for individuals to overcome this anxiety (Schmid et al., 2015). Additionally, one study showed that individuals with lower levels of social support are more likely to develop anxiety and depression (Zhou et al., 2013). College students can receive psychological support from family members, friends, and other sources, and this type of support can serve as positive psychological resources for college students to cope with social anxiety (Liu et al., 2023). Therefore, the existing literature shows that the level of social support is an important factor in college students’ resistance to social anxiety (Vodermaier and Linden, 2019), and social support may play a mediating role in the relationship between other factors and social anxiety (Zhang et al., 2017). Therefore, in order to explore the impact mechanism of physical activity on social anxiety among college students and determine the potential role of social support, this study proposed the second hypothesis:

*H2*: Social support plays a mediating role between physical activity and social anxiety among college students.

### 1.3 The mediating role of psychological capital

Psychological capital refers to a positive psychological state of an individual, which provides corresponding psychological resources for individuals to effectively cope with changes in the complex external environment (Wang et al., 2017). From the perspective of the cognitive-behavioral model of social anxiety and the stress-buffering hypothesis, the causes that affect an individual’s social anxiety usually come from the individual’s perception of whether the social situation is controllable. Positive psychological capital can buffer the individual’s nervousness and negative evaluation in unfamiliar situations. The social anxiety caused makes them adapt to changes in the external environment more quickly (Fu et al., 2012; Heimberg et al., 2014). This suggests that psychological capital may play an important role in alleviating social anxiety. Research shows that positive construction of psychological resources can improve an individual’s perception of the controllability of the environment and events (Hill and Hamm, 2019). Looking back at the definition of social anxiety, the uncontrollability or negative evaluation of the social interaction process is precisely the root cause of social anxiety in individuals. This explains the rationality of psychological capital alleviating social anxiety. Physical activity has been widely recognized as a green and effective means to improve the psychological capital level of college students and ensure their mental health development. Li et al. (2020b) explored the relationship between physical activity, psychological capital and social anxiety among left-behind children and found that physical activity can not only directly affect social anxiety, but also have an indirect effect on social anxiety through the mediating effect of psychological capital. However, whether this relationship exists among college students remains to be explored. Therefore, this study proposes the third hypothesis:

*H3*: Psychological capital plays a mediating role between physical activity and social anxiety among college students.

### 1.4 The chain mediating role of social support and psychological capital

Previous empirical studies have made preliminary explorations into how physical activity affects social anxiety. However, previous studies have focused more on the mediating role of either social support or psychological capital in the relationship between physical activity and social anxiety. There has been no analysis of social support and social anxiety. Psychological capital is also included as a mediating variable in the path model study of the impact of physical activity on social anxiety. The social support buffering theory points out that social support can reduce the stress perceived by individuals and promote their social adaptation and mental health development (Yang et al., 2020; Wang and Li, 2022). This shows that social support can significantly predict an individual’s level of psychological capital. Then, physical activity has a positive impact on an individual’s level of social support, and social support is closely related to psychological capital, and psychological capital can significantly and negatively predict social anxiety. Therefore, this study believes that physical activity is effective in preventing and alleviating social anxiety among college students. There may be an indirect path of physical activity→social support→psychological capital→social anxiety. Therefore, this study proposes the fourth hypothesis:

*H4*: Social support and psychological capital play a chain mediating role between physical activity and social anxiety among college students.

**Figure 1:**
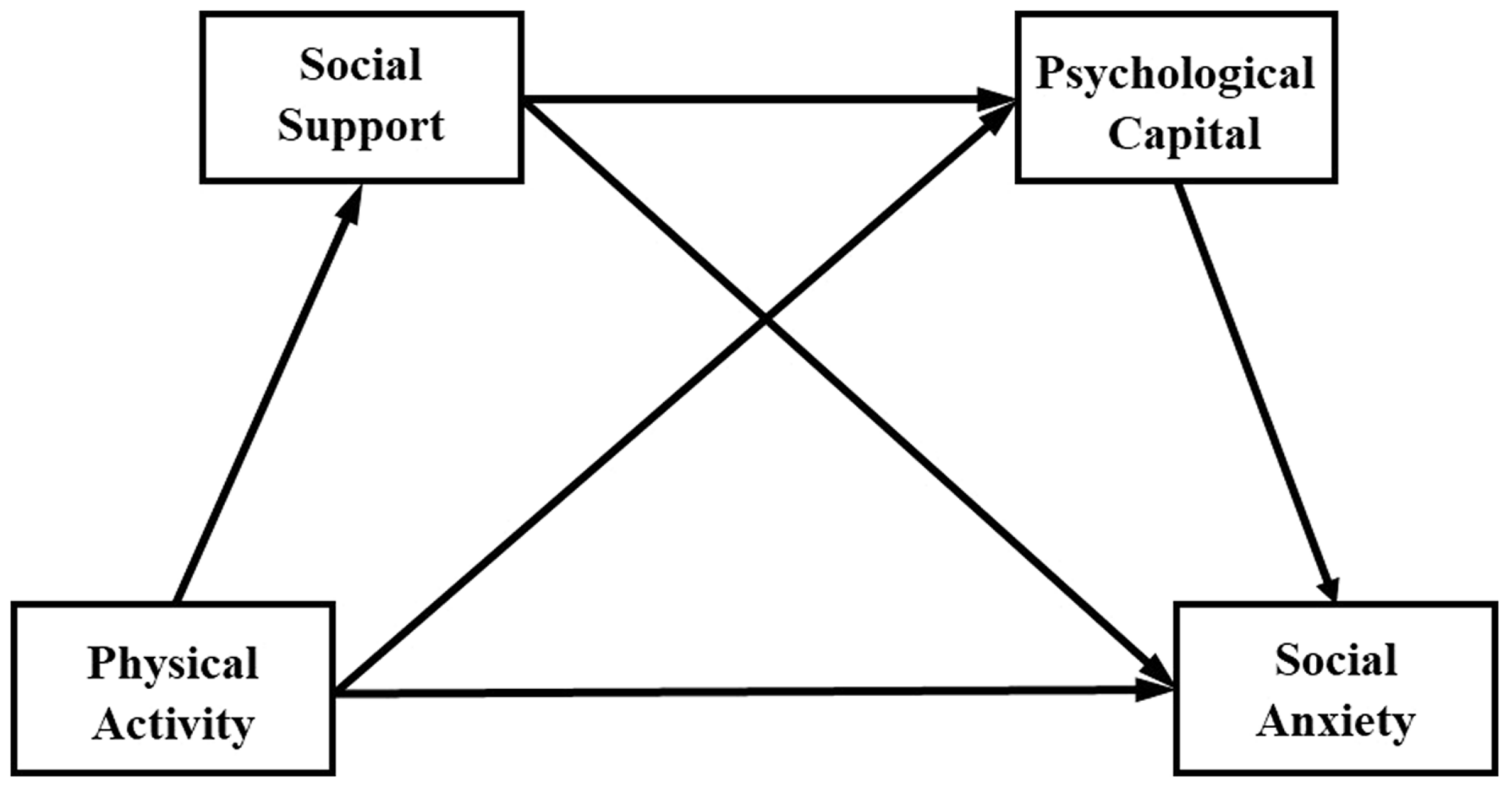
Hypothetical model.

To sum up, this study took college students as the research object to explore the relationship between physical activity, social support, psychological capital and social anxiety among college students. The hypothetical model is shown in Figure 1.

## 2 Materials and methods

### 2.1 Participants

This study used the convenience sampling method to distribute questionnaires offline in 5 universities in Shandong Province. All subjects participated voluntarily, and after obtaining their informed consent, trained staff uniformly guided them in filling out the questionnaires. A total of 1,005 surveys were collected, and subsequently, exclusion criteria were established based on reliability, sensitivity, and operational principles. The criteria for excluding invalid surveys are as follows: Firstly, Incomplete provision of personal basic information. Secondly, Selection of options in questions that entirely lack logical coherence, displaying clear logical errors. Thirdly, More than 80% of responses being identical, indicating a clear pattern of consistent answering. Fourthly, More than 10 instances of missing answers throughout the entire survey. Fifthly, Responses not meeting the specified requirements, demonstrating a phenomenon of answering questions that were not asked. Sixthly, Selecting two or more options for single-choice questions.

After excluding invalid questionnaires based on the above criteria, a total of 874 valid questionnaires were collected (86.96%). Among them, 482 are boys (55.15%) and 392 are girls (44.85%); 587 are only children (67.16%) and 287 are not only children (32.84%); 200 are freshmen (22.88%) and 324 are sophomores (37.07%), 224 juniors (25.63%), and 126 seniors (14.42%).

### 2.2 Measures

#### 2.2.1 Physical activity

Physical activity level was measured using the Physical Activity Rating Scale (PARS-3) (Liang, 1994). which mainly consists of three items: exercise intensity, exercise frequency, and duration of each exercise. The Likert 5-point scoring method was used to calculate the total score. It measures the amount of individual exercise. The higher the score, the greater the amount of physical exercise. The formula for calculating the total score is: physical activity level = exercise intensity × (exercise duration - 1) × exercise frequency. Score range is 0–100. The Cronbach’s α of this scale in this study was 0.896.

#### 2.2.2 Social support

The social support scale developed by Zimet et al. (1988). This scale has 12 items in total, including three dimensions: family support, friend support and other support. It adopts a 5-point Likert scoring method (1 = completely dissatisfied, 5 = completely satisfied). The higher the total score, the better the individual has achieved. The more social support there is. The Cronbach’s α of this scale in this study was 0.838.

#### 2.2.3 Psychological capital

The positive psychological capital scale compiled by Zhang et al. (2010). was used. This scale has a total of 26 items, including 4 dimensions: self-efficacy, optimism, hope, and resilience. It adopts a 7-point Likert scoring method (1 = not at all, 7 = Completely consistent), the higher the total score, the higher the level of psychological capital of the individual. The Cronbach’s α of this scale in this study was 0.934.

#### 2.2.4 Social anxiety

Social anxiety uses the Social Anxiety Scale developed by American psychologist La Greca and Lopez (1998). Chinese scholar Zhu Haidong translated and revised the scale in 2008 and has been widely used in related research in the Chinese cultural context. This scale has 13 items in total, including fear of negative evaluation, social avoidance and annoyance in general/unfamiliar environments. It adopts a 5-point Likert scoring method (1 = completely inconsistent, 5 = completely consistent). The higher the total score, the higher the total score. The more severe the individual’s social anxiety is. The Cronbach’s α of this scale in this study was 0.947.

### 2.3 Data analysis

First, SPSS 20 was used to conduct descriptive statistics, reliability and validity testing, correlation analysis, and regression analysis on the data. Secondly, the mediation effect was analyzed through Model 6 in the SPSS process 3.3 macro program compiled by Hayes, and the Bootstrap (5,000 times) method was used to test the mediation effect.

## 3 Results

### 3.1 Common method bias tests

Because this study uses offline self-reporting to collect data, it is necessary to conduct a common method bias test on the relevant data. First, some procedures such as reverse scoring items and anonymous measurement were set up during the testing process to reduce common method bias. Secondly, Harman’s single factor test was used to check common method bias. The results showed that a total of 10 factors had characteristic roots greater than 1, and the variance explanation rate of the largest common factor was 18.64%, which was far lower than the critical value of 40%, indicating that this study was not sufficient. There is significant common method bias (Podsakoff et al., 2003).

### 3.2 Descriptive statistics and correlations

Pearson correlation analysis was used to explore the correlation between physical activity, social support, psychological capital and social anxiety. As shown in Table 1, there is a significant positive correlation between college students’ physical activity, social support, and psychological capital; there is a significant negative correlation between college students’ social anxiety and physical activity, social support, and psychological capital.

**Table 1:** Descriptive statistics and related analysis of each variable.

Variable	^−^*x* ± *s*	PA	SS	PC	SA
PA	21.44 ± 21.80	–			
SS	39.62 ± 7.94	0.354**	–		
PC	89.66 ± 19.12	0.448**	0.556**	–	
SA	34.93 ± 14.01	−0.326**	−0.382**	−0.432**	–

PA, Physical Activity; SS, Social Support; PC, Psychological Capital; SA, Social Anxiety; *p < 0.05; **p < 0.01; ***p < 0.001.

### 3.3 The mediating effects analysis

Research has shown that gender and age can influence psychological health issues such as anxiety and stress among college students (Liu et al., 2023). After setting gender and grade as control variables, the mediating effects of social support and psychological capital on the relationship between physical activity and social anxiety among college students were tested. Model 6 in the SPSS process 3.3 macro program compiled by Hayes was used to repeat sampling 5,000 times and calculate the 95%CI at the same time. The regression results are shown in Table 2: Physical activity of college students can significantly negatively predict social anxiety (β = −0.314, *p* < 0.001), significantly positively predict social support (β = 0.205, *p* < 0.001) and psychological capital (β = 0.205, *p* < 0.001) (β = 0.185, *p* < 0.001); social support can significantly and positively predict psychological capital (β = 0.507, *p* < 0.001); when physical activity, social support and psychological capital simultaneously predict social anxiety, social support (β = −0.311, *p* < 0.001) and psychological capital (β = −0.395, *p* < 0.001) can both significantly and negatively predict social anxiety. At this time, the negative prediction of physical activity on social anxiety is still significant (β = −0.136, *p* < 0.001), which indicates that the chain mediating role of social support and psychological capital between physical activity and social anxiety among college students is established.

**Table 2:** Regression analysis of chain mediation model of social support and psychological capital.

Outcome variable	Predictor variable	*R*	*R* ^2^	*F*	β	*t*
SA	PA	0.329	0.108	35.273***	−0.314	−10.156***
	Gender				−0.096	−1.354
	Grade				−0.005	−0.134
SS	PA	0.356	0.126	41.958***	0.205	11.192***
	Gender				−0.044	−1.041
	Grade				0.011	0.503
PC	PA	0.622	0.387	136.941***	0.185	10.110***
	SS				0.507	16.041***
	Gender				0.085	2.159**
	Grade				0.030	1.521
SA	PA	0.483	0.233	52.796***	−0.136	−4.185***
	SS				−0.311	−5.160***
	PC				−0.395	−6.937***
	Gender				−0.085	−1.286
	Grade				0.013	0.378

PA, Physical Activity; SS, Social Support; PC, Psychological Capital; SA, Social Anxiety; *p < 0.05; **p < 0.01; ***p < 0.001.

**Figure 2:**
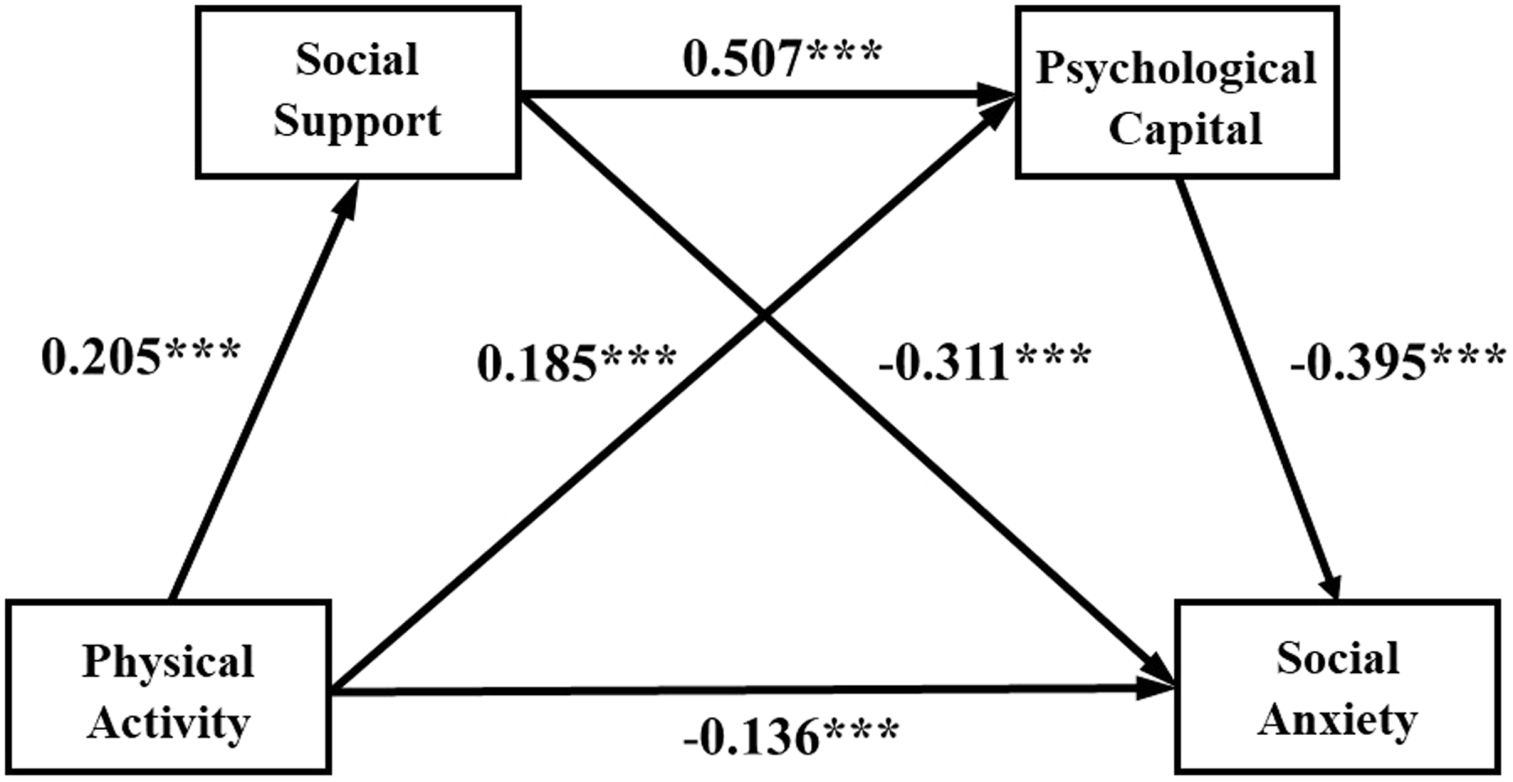
Chain mediation model.

After further conducting mediation effect analysis, the results are shown in Table 3 and Figure 2. The direct effect of physical activity on social anxiety among college students is −0.136, and the confidence interval does not include 0, indicating that the direct effect is significant, accounting for 43.31% of the total effect; the total indirect effect value is −0.178, and the confidence interval does not include 0, indicating that social support and the mediating effect of psychological capital between college students’ physical activities and social anxiety is significant. Accounting for 56.68% of the total effect. Among them, the path effect value using social support as the mediating variable is −0.064, 95%CI = [−0.093, −0.039]; the path effect value using psychological capital as the mediating variable is −0.073, 95%CI = [−0.101, −0.048]; while using social support and psychological capital as mediating variables, the chain mediation path effect value is −0.041, 95%CI = [−0.056, −0.027]. It can be seen that the above three indirect effect paths are all significant.

**Table 3:** Analysis of the mediating effects of body image and psychological capital.

Model effect	Effect size	Boot SE	95%CI		Effect proportion
			LLCI	ULCI	
Total effect	−0.314	0.031	−0.374	−0.253	
Direct effect: PA → SA	−0.136	0.032	−0.199	−0.072	43.31%
Total indirect effect	−0.178	0.018	−0.215	−0.143	56.68%
PA → SS → SA	−0.064	0.014	−0.093	−0.039	20.38%
PA → PC → SA	−0.073	0.013	−0.101	−0.048	23.25%
PA → SS → PC → SA	−0.041	0.007	−0.056	−0.027	13.05%

PA, Physical Activity; SS, Social Support; PC, Psychological Capital; SA, Social Anxiety.

## 4 Discussion

### 4.1 The impact of physical activity on social anxiety among college students

The results of the study found that physical activity was significantly negatively correlated with college students’ social anxiety, and after incorporating mediating variables, the negative predictive effect of physical activity on college students’ social anxiety was still significant, that is, as the amount of activity increased, the level of social anxiety increased significantly. Decrease, assuming H1 is true. The results of this study are consistent with previous studies (Brunet and Sabiston, 2009). Their study showed that the amount and time of exercise are positively related to the mental health of adolescents, and after controlling for gender variables, physical activity can significantly reduce social anxiety. Physical exercise is an important part of college students’ campus life. Regular participation in sports can improve self-confidence and ability to resist frustration. The cooperation and communication brought about by team sports can encourage individuals to acquire good social skills, thereby improving social abilities to maintain appropriate interpersonal relationships (Yang and Xiang, 2021). The occurrence of social anxiety is the comprehensive result of the interaction of internal and external factors related to the individual. When an individual is unable to handle emergencies and lacks effective means to deal with negative evaluations, social anxiety manifestations such as social avoidance and social phobia are likely to occur. People who often participate in sports Active college students have strong psychological resilience and perseverance, which are enough to resist the intrusion of social anxiety triggers from internal and external sources, and prevent and alleviate the occurrence of social anxiety.

### 4.2 Separate mediating effects of social support and psychological capital

The research results show that physical activity not only directly and negatively predicts social anxiety among college students, but also has an indirect impact on social anxiety through the mediating role of social support and psychological capital. Hypotheses H2 and H3 are established. Physical activity reduces social anxiety among college students by increasing their levels of social support, a result consistent with existing research (Calsyn et al., 2005). Research also indicates that positive peer relationships provide important social support for college students in their academic and personal lives. According to the ecological systems theory of human development, family and peers are two significant systems influencing an individual’s development. If one system is no longer as active, other systems will compensate and play a more crucial role (Hertler et al., 2018; Liu et al., 2023). Newly-adult college students often have reduced contact with their parents after entering university, resulting in decreased psychological support from the family. Therefore, peer relationships become a crucial assurance for them to reduce social anxiety (Cavanaugh and Buehler, 2016). Physical exercise can exactly increase the opportunities for college students to interact with other peers. For example, team sports activities not only provide good training opportunities for cultivating college students’ interpersonal communication skills, but also provide opportunities for developing high-quality peer relationships and obtaining more social support. Provides favorable conditions, thereby reducing an individual’s level of social anxiety (Baskin et al., 2015). This result provides a new perspective for exploring the impact of physical activity on social anxiety, suggesting the important role of social support as a mediating variable in alleviating social anxiety among college students.

Psychological capital is an important protective factor for college students’ emotional problems (Kwok et al., 2015). Good psychological quality is an important prerequisite for college students to reduce their susceptibility to negative evaluations from all aspects, and psychological capital is an important part of this quality (Fan et al., 2018b). Self-efficacy in psychological capital can help enhance individual self-confidence and reduce the negative interference from external negative evaluations (Fan et al., 2018a); optimism and resilience are important personality traits that affect social anxiety (Dolcos et al., 2016), which can help college students find ways to vent their emotions in a timely manner and resolve problems when social anxiety occurs. The sense of discomfort is to enhance psychological recovery (Stanley and Mettilda Bhuvaneswari, 2016); the sense of hope starts from eliminating the sense of helplessness and fear in the process of social interaction, helping individuals to establish communication expectations and establish social interaction expectations (Peh et al., 2017). In real life, not only do the external environment and personal psychological traits play a regulatory role in social anxiety respectively, but personal psychological traits often act as mediating variables. For example, physical activity can help individuals obtain high-quality peer relationships to obtain more psychological support, improve psychological capital levels, and thereby reduce social anxiety (Seabrook et al., 2016). This shows that psychological capital plays an obvious mediating and supporting role as an important coping resource to resist negative feedback and social pressure in the intrinsic mechanism of physical activity affecting social anxiety.

### 4.3 The chain mediating role of social support and psychological capital

In addition, this study also found that social support and psychological capital play a chain mediating role between physical activity and social anxiety among college students, and hypothesis H4 is established. Social support can positively predict psychological capital. Compared with low levels of social support, individuals with high levels of social support show higher levels of psychological capital, which is consistent with the results of this study (Zhao, 2023). On the one hand, the main mechanism through which psychological capital affects individual social anxiety is to comprehensively improve the individual’s ability to offset the influence of adverse factors in the social process from the four aspects of self-efficacy, optimism, resilience and hope, and reduce the probability of social distress. In this process, social support, as an important part of self-cognition evaluation, plays a key role in building a high level of psychological capital. On the other hand, an individual’s self-understanding not only comes from self-examination, but also from feedback during interactions with others. Insufficient interaction or feedback errors will lead to limitations in individual self-understanding, resulting in self-doubt and further development. For socially anxious behaviors. Bias theory suggests that when performing physical activities, individuals can pay more attention to their own health and external image to control the impression they leave on others while meeting their needs for interaction with the outside world, and quickly cultivate positive perceptions of social support. Do your best to correct cognitive biases and self-doubt to maintain high levels of psychological capital to deal with social anxiety (Li and Feng, 2013; Li et al., 2015). Therefore, including social support and psychological capital as chain mediating variables in the study provides a new perspective for exploring the potential mechanism by which physical activity affects social anxiety among college students.

## 5 Research significance, limitations and future research

Although existing research has achieved certain results on how physical activity affects social anxiety, most scholars prefer to examine the single role of body image or psychological capital. This study incorporates social support and psychological capital into the path model of the impact of physical activity on social anxiety. Based on empirical testing, it attempts to discuss the complex effects of physical activity, social support, and psychological capital on preventing and alleviating social anxiety. It not only provides Further exploring the mechanism by which physical activity reduces social anxiety among college students provides empirical reference and theoretical support. It also has certain practical significance in guiding college students to pay attention to perceived social support and improve their psychological capital levels to cope with social anxiety.

In summary, after clarifying the relationship between physical activity, social support, psychological capital, and social anxiety among college students, this study proposes corresponding suggestions from the following three aspects.

Firstly, emphasize the significant role of various forms of physical activity in addressing mental health issues among college students. Individuals should maintain a positive and proactive attitude in their college lives, regulating their life and work rhythm by engaging in activities such as joining sports clubs, participating in travel activities, and taking part in sports competitions to avoid falling into self-isolation and self-doubt. Particularly, it is essential to emphasize participation in team sports activities, such as group cycling, basketball and soccer matches, and group hiking. Team sports activities can serve as an important means of communication and interaction with the outside world, helping college students enrich their leisure time, build social networks, and overcome mental health issues such as social anxiety.

Secondly, emphasize the critical role of internal cognitive factors such as social support and psychological capital in addressing mental health issues among college students. External interventions are undoubtedly important, but the ultimate effectiveness depends on internal cognition. Therefore, college students can record their daily work and life experiences and carefully reflect on them. Through reflection and meditation, they can engage in self-psychological health education and strengthen their psychological resilience, continuously cultivating positive psychological capital such as perseverance, optimism, and self-efficacy. This not only greatly benefits individual growth but also enhances the effectiveness of external interventions, further alleviating social anxiety and improving mental health among individuals.

Thirdly, be proficient in utilizing advanced scientific technologies such as artificial intelligence to conduct more precise screening and prevention of mental health issues among college students. With the rapid development of the internet, an increasing number of artificial intelligence online technologies are being applied in the quality of college students’ mental health, including some technologies combined with cognitive behavioral therapy. For example, applications like MoodKit, MoodPrismying, etc., can provide users with instant interventions based on cognitive behavioral therapy through smartphones, thereby enhancing users’ subjective well-being and effectively alleviating mental health problems including social anxiety. College students, as the most open-minded group, can conduct self-diagnosis and early intervention through such advanced information technologies, preventing and overcoming potential mental health risks.

Moreover, schools and society should take strong measures to conduct activities such as college students’ mental health self-examination, identify college students with mental health issues, and provide personalized interventions and treatments based on their specific situations.

However, this study still has the following shortcomings: First, the sources of subjects are too single. Future research should expand the sample scope to verify the generalizability of the research results; second, the mediating variables are not fully included, and the explanation for physical activity’s reduction of social anxiety is limited. Future research should consider more different predictor variables to further reveal the potential paths through which physical activity affects social anxiety; third, this study is a cross-sectional study, and longitudinal follow-up experiments are needed in the future to study the causal relationship between the variables. Multi-level and comprehensive exploration of the mechanism of physical activity in preventing and alleviating social anxiety among college students.

## 6 Conclusion

This study aimed to examine the relationship between physical activity, social support, psychological capital and social anxiety, especially whether social support and psychological capital mediate the relationship between physical activity and social anxiety. Finally, the following conclusions are drawn: (1) Physical activity can reduce and alleviate the social anxiety problem of college students. (2) Social support and psychological capital not only play a separate mediating role between physical activity and social anxiety, but also have a chain mediating role. Therefore, being actively engaged in physical activity will be one of the effective means for college students to effectively overcome the problem of social anxiety, and it can also help them develop healthy exercise habits in their future lives. It is worth noting that the results of this study should be proven through other cross-sectional or longitudinal studies, or even experimental studies, to fully ensure its reliability.

## Data availability statement

The original contributions presented in the study are included in the article/supplementary material, further inquiries can be directed to the corresponding author.

## Ethics statement

Ethical review and approval was not required for the study on human participants in accordance with the local legislation and institutional requirements. Written informed consent from the patients/participants or patients/participants’ legal guardian/next of kin was not required to participate in this study in accordance with the national legislation and the institutional requirements.

## Author contributions

YD: Writing – original draft, Visualization, Software, Investigation, Formal analysis, Data curation, Conceptualization. XW: Writing – review & editing, Validation, Supervision, Resources, Project administration, Methodology, Funding acquisition.
